# The construction, validation and promotion of the nomogram prognosis prediction model of UCEC, and the experimental verification of the expression and knockdown of the key gene GPX4

**DOI:** 10.1016/j.heliyon.2024.e24415

**Published:** 2024-01-20

**Authors:** Lindong Zhang, Jialin Wang, Yan Guo, Haodi Yue, Mengjun Zhang

**Affiliations:** a Department of Gynecology, The Third Affiliated Hospital of Zhengzhou University, 7 Rehabilitation Front Street, Zhengzhou, 450052, China; b Department of Orthopedics, Xuanwu Hospital, Capital Medical University, Beijing, 100000, China; c Department of Center for Clinical Single Cell Biomedicine, Henan Provincial People's Hospital, People's Hospital of Zhengzhou University, No. 7 Weiwu Street, Zhengzhou, 450003, China; d Department of Oncology, Henan Provincial People's Hospital, People's Hospital of Zhengzhou University, No. 7 Weiwu Street, Zhengzhou, 450003, China

**Keywords:** Uterine corpus endometrial carcinoma, Pyroptosis-related genes, GPX4, Prognostic prediction model, Web-based dynamic nomogram

## Abstract

**Background:**

Adequate prognostic prediction of Uterine Corpus Endometrial Carcinoma (UCEC) is crucial for informing clinical decision-making. However, there is a scarcity of research on the utilization of a nomogram prognostic evaluation model that incorporates pyroptosis-related genes (PRGs) in UCEC.

**Methods:**

By analyzing data from UCEC patients in the TCGA database, four PRGs associated with prognosis were identified. Subsequently, a “risk score” was developed using these four PRGs and LASSO. Ordinary and web-based dynamic nomogram prognosis prediction models were constructed. The discrimination, calibration, clinical benefit, and promotional value of the selected GPX4 were validated. The expression level of GPX4 in UCEC cell lines was subsequently verified. The effects of GPX4 knock-down on the malignant biological behavior of UCEC cells were assessed.

**Results:**

Four key PRGs and a “risk score” were identified, with the “risk score” calculated as (−0.4323) * GPX4 + (0.2385) * GSDME + (0.0525) * NLRP2 + (−0.3299) * NOD2. The nomogram prognosis prediction model, incorporating the “risk score,” “age,” and “FIGO stage,” demonstrated moderate predictive performance (AUC >0.7), good calibration, and clinical significance for 1, 3, and 5-year survival. The web-based dynamic nomogram demonstrated significant promotional value (https://shibaolu.shinyapps.io/DynamicNomogramForUCEC/). UCEC cells exhibited abnormally elevated expression of GPX4, and the knockdown of GPX4 effectively suppressed malignant biological activities, including proliferation and migration, while inducing apoptosis. The findings from tumorigenic experiments conducted on nude mice further validated the results obtained from cellular experiments.

**Conclusion:**

Following validation, the nomogram prognosis prediction model, which relies on four pivotal PRGs, demonstrated a high degree of accuracy in forecasting the precise probability of prognosis for patients with UCEC. Additionally, the web-based dynamic nomogram exhibited considerable potential for promotion. Notably, the key gene GPX4 exhibited characteristics of a potential oncogene in UCEC, as it facilitated malignant biological behavior and impeded apoptosis.

## Highlights

• The nomogram clinical prediction model can intuitively predict the diagnosis or prognosis.
• A nomogram was established by PRGs and clinical features, and its predictive performance and promotion value were validated.
• The key gene (GPX4) was selected for validation, ultimately providing an accurate option for prognostic assessment of UCEC.

## 1 Introduction

Uterine Corpus Endometrial Carcinoma (UCEC) ranks as the second most prevalent malignant neoplasm within the female reproductive system in China, while attaining the highest incidence in developed nations [1,2]. Regrettably, patients afflicted with advanced, recurrent, or distant metastatic UCEC exhibit a dismal prognosis, with a 5-year survival rate below 20 % [3]. To enhance the outlook for this condition, researchers are diligently undertaking diverse endeavors. For instance, it is widely acknowledged that personalized prognostic assessment and appropriate adjuvant therapeutic alternatives can be conducted by leveraging diverse clinical attributes and genetic sequencing of pathological specimens obtained post-malignant tumor excision. This holds immense importance in enhancing patient prognosis [4,5].

Various clinical prediction models, including logistic regression models, Cox regression models, machine learning models, deep learning models, and nomogram models, have demonstrated remarkable predictive capabilities in diagnosing and prognosing diseases [[6], [7], [8], [9], [10], [11], [12]]. Notably, the nomogram model stands out for its intuitive and straightforward nature, enabling the creation of easily disseminated web-based dynamic nomograms via network platforms. This advancement greatly facilitates disease prognosis prediction, clinical decision-making, and ultimately the translation of research findings into clinical practice [13]. For instance, a study successfully demonstrated the ability to predict and evaluate the prognosis of myometrial invasion in patients with early-stage endometrial cancer using radiomics and nomograms [14]. In the case of UCEC, constructing a nomogram prognosis prediction model based on the patient's clinical, pathological, and tumor gene characteristics is a significant undertaking and may help to use the predicted survival probability to develop personalized treatment plans and clinical decisions for patients. Currently, there is a dearth of pertinent, sound, and exemplary research studies.

Pyroptosis, as a programmed cell death mechanism, is an intrinsic component of the cellular cycle and biological processes [15,16]. Notably, contemporary research has established a strong correlation between pyroptosis and the pathogenesis and progression of numerous diseases, particularly cancer [[17], [18], [19], [20]]. Extensive evidence supports the notion that pyroptosis exerts a significant influence on the proliferation, invasion, and metastasis of diverse cancer cell types, such as those found in kidney, colorectal, lung, and ovarian cancers, ultimately resulting in unfavorable prognoses [[21], [22], [23], [24]]. However, it is worth mentioning that certain investigations have also indicated that pyroptosis possesses a dual nature in the context of cancer. Certain pyroptosis-related genes (PRGs) have been found to facilitate tumor growth by providing nutrients, while in certain instances, other PRGs have demonstrated the ability to inhibit tumor initiation and progression. Although the mechanisms involved are intricate, they warrant further investigation [25]. Nevertheless, there is a scarcity of prognostic prediction models for UCEC that are based on PRGs, despite their significant potential and research significance.

This study aims to identify prognostically relevant PRGs and employ the LASSO method to develop risk scores, nomograms, and a dynamic web-based nomogram that can effectively predict the prognosis of UCEC. Additionally, this study seeks to investigate the involvement of the key gene, GPX4, in UCEC through rigorous foundational experiments.

## 2 Materials and methods

1. Clinical informpation and gene expression profiles of the patients

The TCGA database provided the data for 542 UCEC samples and 35 normal control samples, encompassing various clinical characteristics, survival data, and gene expression profiles. These fundamental data were utilized for subsequent key pyroptosis-related gene (PRG) screening, the comprehensive prognostic analysis of key PRGs, mutation-related analysis, the construction and validation of prognostic prediction models, Gene Set Enrichment Analysis (GSEA), Immune Microenvironment Analysis, Gene Ontology (GO), and KEGG analyses, among others.

2. Screening and prognostic evaluation of four key PRGs

Initially, a comprehensive collection of 33 PRGs was obtained from relevant literature reviews. Subsequently, the expression levels of these 33 PRGs were examined in both UCEC and adjacent normal tissues. The prognostic significance of these PRGs in UCEC patients was assessed through univariate and multivariate Cox regression analyses, as well as survival analysis, leading to the identification of four key PRGs. Furthermore, Spearman's correlation analysis was employed to investigate the relationship between these 4 key PRGs, microsatellite instability (MSI), and tumor mutation burden (TMB) in UCEC. The protein expression of key PRGs was also validated through the immunohistochemical findings from The Human Protein Atlas.

3. Construction, validation, and promotion of a nomogram prognosis prediction model based on four key PRGs

Following the identification of four significant PRGs for prognosis evaluation, a machine learning technique known as LASSO Cox regression analysis was employed to establish a “risk score” based on the expression levels of these four PRGs, as indicated by the equation: “risk score” = (−0.4323) * GPX4 + (0.2385) * GSDME + (0.0525) * NLRP2 + (−0.3299) * NOD2. The distributions of the “risk score,” survival status, and gene expression levels were presented. The study also demonstrated variations in the “risk score” across different subgroups based on clinical features. Subsequently, UCEC patients were categorized into a “high-risk group” and a “low-risk group” based on their respective “risk scores.” Subsequent analyses, including survival analysis, ROC curve analysis, and Cox regression analysis, were conducted to validate the prognostic significance of the aforementioned “risk score.”

Subsequently, adhering to the guidelines for developing nomogram clinical prediction models [26], in conjunction with the findings derived from Cox regression analysis, the selection of “risk score,” “Age,” and “Figo stage” as predictors was determined. Consequently, an ordinary nomogram prognosis prediction model was constructed to estimate the precise likelihood of survival beyond 1, 3, and 5 years for patients diagnosed with Uterine Corpus Endometrial Carcinoma (UCEC). Subsequently, the Receiver Operating Characteristic curve (ROC curve), Area Under ROC Curve (AUC), calibration curve, Decision Curve Analysis (DCA), Area Under Decision Curve (AUDC), and other metrics were employed to assess the prognostic predictive model's performance and scientific validity at 1 year, 3 years, and 5 years intervals. The ROC curve and AUC value were utilized to evaluate the model's discriminatory ability, with an AUC value exceeding 0.7 indicating moderate predictive value and discrimination. The calibration curve was employed to verify the calibration of the model, and the model's calibration was deemed satisfactory when the “model line” aligned with the “Ideal line”. The DCA curve and AUDC value were utilized to assess the clinical advantages of the model. When compared to the “All line” and the “None line,” the model outperformed them under a specific decision threshold. In other words, a higher AUDC value indicated superior clinical benefits of the model. In this study, based on shinyapp (https://www.shinyapps.io/), a web-based dynamic nomogram (https://shibaolu.shinyapps.io/DynamicNomogramForUCEC/) was developed to create personalized survival curves for UCEC patients and accurately predict survival probabilities within specific time frames. This tool has the potential to greatly facilitate clinical translation and is highly advantageous.

## 3 Cell culture

The human endometrial cells (HEEC) and endometrial cancer cells (HEC-1A, HEC-1B, and RL95-2) used in this research were obtained from The Cell Bank of Type Culture Collection of The Chinese Academy of Sciences. The cells were cultured in incubators at a temperature of 37 °C and a carbon dioxide concentration of 5 %. The culture media used were DMEM, MoCoy's 5A, and MEM, supplemented with 10 % FBS from Thermo Fisher Scientific.

## 4 RT-qPCR

Total RNA was extracted from the respective cell lines. Subsequently, the concentration and purity of the total RNA were assessed using a NanoDrop One spectrophotometer from Thermo Fisher Scientific. Reverse transcription was then conducted to generate cDNA using Novoprotein. Finally, the expression levels of key genes were determined through RT-qPCR using NovoStart SYBR qPCR SuperMix Plus from Novoprotein. The thermal cycling conditions consisted of an initial denaturation step at 95 °C for 10 min, followed by denaturation at 95 °C for 10 s, and annealing and extension at 60 °C for 30 s. This process was repeated for a total of 40 cycles. The relative expression level of the gene was determined using the 2^−ΔΔCT^ method. The average gene expression levels were compared using a *t*-test.

## 5 TUNEL assay

Apoptosis was detected using immunofluorescent staining, which was performed following the steps outlined in the Tunel kit. DAPI was utilized as a counterstain to identify the nuclei of the cells. The stained cells were observed using an immunofluorescence microscope (BX60; Olympus) within three non-overlapping fields of view.

## 6 Clone formation assay

The concentration of cells was 200 cells per well. Each cell line was individually interfered with using si-GPX4, and each well was repeated three times. Colony formation was monitored under the microscope every 7 days, with the medium being replenished weekly. Formed colonies were stained with 4 % paraformaldehyde and 0.1 % crystal violet for a duration of 20 min, followed by gentle washing with ddH_2_O and subsequent microscopic photography.

## 7 Wound healing assay

Cells transfected with si-GPX4 were inoculated onto 6-well plates at a fusion rate of 80 %. A 200-μL pipette was employed to create a linear scratch along the central region of the plate. The width of the scratch was captured via microscopy at 0 and 48 h, and subsequently subjected to analysis using ImageJ v1.8.0 software.

## 8 Transwell experiment

Cells that had been transfected with si-GPX4 were introduced into 200 μL of serum-free medium. Concurrently, 700 μL of medium containing 15 % FBS was introduced into the lower chamber. After 24 h, the cells were fixed using 4 % paraformaldehyde and stained with 0.1 % crystal violet for 20 min. Finally, three random areas were photographed, and the cells were quantified and measured.

### 8.1 Ki67 immunofluorescence assay

The staining procedure described in the Ki-67 kit was employed to perform the staining. DAPI was utilized to stain the cell nucleus. The staining was observed using an immunofluorescence microscope (BX60; Olympus, Tokyo, Japan) in three randomly selected fields of view.

## 9 Flow cytometry for apoptosis analysis

Apoptosis was detected using the Annexin V FITC Apoptosis Kit (BD Pharmingen, San Diego, CA, USA) and flow cytometry (Accuri C6; BD Biosciences, San Jose, CA, USA). The resulting data were processed using Flow JO v10.5.

## 10 Western blotting assay

The Western blotting assay was conducted to determine the protein levels of GPX4 in various cell lines. The levels of validated proteins for GPX4 knockdown were assessed through various experimental procedures, including gel preparation, sample addition, electrophoresis, and membrane transfer. Subsequently, primary and secondary antibodies were incubated, and chemiluminescence was developed.

## 11 CCK-8 experiment

Si-GPX4 cells were cultured in 96-well plate dishes following the protocol outlined in the CCK-8 (Abmole Bioscience, Houston, TX, USA) kit. The optical density (OD) of each well was measured at 450 nm using a universal microplate spectrophotometer (ENsight; PerkinElmer, Waltham, MA, USA). The obtained data were analyzed using GraphPad Prism (v9.0).

## 12 In vivo tumor xenograft experiments

Six female C-NKG mice (Saiye Suzhou Biotechnology Co., Ltd.), with severe immunodeficiency, were used in this study. The mice were 4 weeks old and weighed approximately 16–18 g. They were kept in a strictly aseptic environment during feeding and handling. HEC-1B cells, which exhibited optimal growth characteristics, and cells transfected with si-GPX4 were selected at a density of 4 × 10^5^/μl. The animal operating table underwent thorough sterilization, and the surgical instruments and equipment were meticulously disinfected and sterilized. Subsequently, the mice were partitioned into two cohorts, each comprising five mice, and were administered 200 μl of HEC-1B cells and si-GPX4 transfected cells. The cell suspension was injected into the dorsal region of the mice's right side. Throughout a span of approximately two weeks, the mice were meticulously monitored, with their body weight and the dimensions (length, L, and width, W) of the subcutaneously transplanted tumors being measured on a weekly basis. After the removal of the tumors, their weight (g), length (L), and width (W) were measured and recorded via photography. Tumor volume was calculated using formula V = L × W [2] × 0.5. This study was reviewed and approved by [Animal Ethics Committee of Zhengzhou University], with the approval number: [No.20230318]. And the study has been carried out in accordance with either the U.K. Animals (Scientific Procedures) Act, 1986 and associated guidelines.

## 13 Statistical analysis

All statistical analyses were conducted using R software (v4.0.2) and GraphPad Prism (v9.0). The pertinent codes for constructing, validating, and generalizing the significant nomogram prognostic prediction model were presented in Supplementary Material 1. The R packages utilized in this study for the development, validation, and dissemination of the nomogram prognostic prediction model encompass “limma,” “glmnet,” “rms,” “timeROC,” “ggDCA,” “regplot,” and “DynNom.” To construct the “risk score” and its corresponding formula, LASSO Cox regression, a machine learning technique, was employed, relying on four pivotal genes. The comparison of group differences in this investigation predominantly relied on the application of Student's T-test and Mann-Whitney test. Various research methods, including Kaplan-Meier survival analysis, univariate and multivariate Cox regression, ROC curve analysis, calibration curve assessment using the Hosmer-Lemeshow goodness of fit test, and DCA curve analysis, were employed to assess the prognosis and validate the efficacy of the nomogram-based prognosis prediction model.

## 14 Results

1. Screening of key prognostic pyroptosis-related genes (PRGs) and their prognostic value

**Fig. 1:**
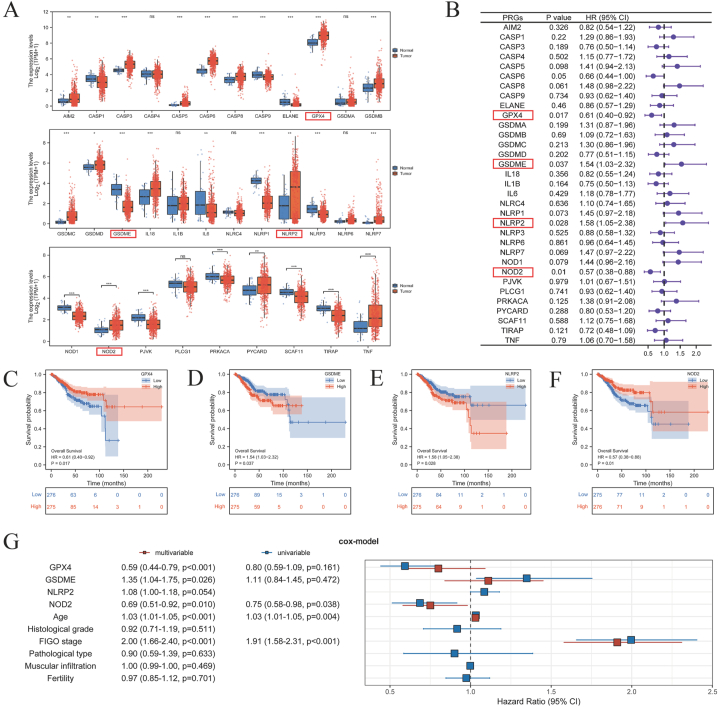
Screening of prognostic-related PRGs based on comprehensive prognostic analysis. (A**)** Gene expression box plots of 33 PRGs in UCEC patients (N = 552) and control adjacent normal group (N = 35) from the TCGA database; **(B)** Univariate Cox regression analysis of OS for each PRG; **(C–F)** Kaplan-Meier survival analysis plots showing the effect of 4 key PRGs (GPX4, GSDME, NLRP2, and NOD2) expression levels on overall survival prognosis; **(G)** Univariate and multivariate Cox regression analysis of OS for 4 key PRGs and clinical factors.

Firstly, the primary concepts and procedures of this investigation were presented in Fig. S6 and Fig. S7. Utilizing a dataset comprising 542 UCEC samples and 35 normal control samples from TCGA, the relative expression levels of 33 PRGs were examined. The findings depicted in Fig. 1A indicated that 27 PRGs exhibited differential expression. The raw data of 542 UCEC samples are recorded in Supplementary Material 2. Subsequently, the results presented in Fig. 1B–F demonstrated that GPX4, GSDME, NLRP2, and NOD2 held promise as prognostic factors in UCEC, thus establishing them as the four key prognostic genes. Additionally, Fig. S1 suggested a potential association between these four specific genes and MSI and TMB in UCEC, as well as a potential presence of gene mutations. However, the univariate and multivariate Cox regression results of the clinical factors with the four key prognostic genes in Fig. 1G were not as ideal as expected. The utilization of these four key prognostic factors alone for prognostic evaluation was not straightforward and direct, thereby indicating the necessity for employing a more sophisticated and scientific approach to elucidate the intricate relationship among them.

2. The construction of the “risk score” based on LASSO Cox and the construction of the nomogram prognosis prediction model

Given the limitations of the common method in assessing the prognostic value of the four key prognostic PRGs, and the effectiveness of the LASSO Cox's machine learning method in analyzing complex correlations in large samples, our study aims to utilize the LASSO Cox's method to investigate the potential prognostic value of these four key PRGs.

**Fig. 2:**
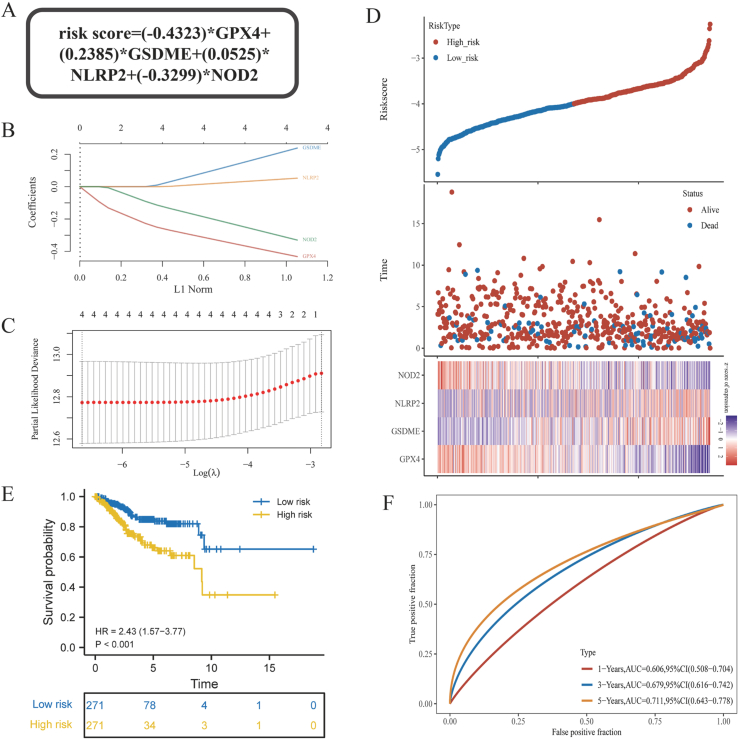
Construction and prognostic significance of the risk score based on 4 key PRGs. (**A)** Calculation formula of the risk score based on LASSO Cox. The risk score was calculated according to the expression levels and specific coefficients of the 4 key PRGs; **(B–C)** The coefficients of the 4 key PRGs and the lambda parameters of LASSO Cox; **(D)** The distribution of the risk score, survival status, and the expression levels of the 4 key PRGs in UCEC patients; **(E)** Overall survival curves of the high and low risk score; **(F)** ROC curves of the risk score for the prognosis of 1-year, 3-year, 5-year survival.

Initially, we constructed a “risk score” incorporating the four key prognostic PRGs using the LASSO Cox approach. The calculation formula for this “risk score” is presented in Fig. 2A as follows: “risk score” = (−0.4323) * GPX4 + (0.2385) * GSDME + (0.0525) * NLRP2 + (−0.3299) * NOD2. Additionally, Fig. 2B and C illustrated the variation of LASSO Cox's penalty coefficient and factor coefficient. Fig. 2D displayed the distributions of the “risk score,” survival status, and gene expression in relation to each other. Furthermore, Fig. S2 illustrated the variations in “risk score” among subgroups of UCEC patients with different clinical factors. Subsequently, UCEC patients were categorized into a “high-risk group” and a “low-risk group” based on their respective “risk score”, and their prognostic value was subsequently investigated. The findings from the survival analysis depicted in Fig. 2E indicated that the overall survival time was notably shorter in the “high-risk group.” Fig. 2F demonstrated that the “risk score” exhibited moderate predictive efficacy for the overall survival of UCEC patients over a span of five years, but displayed less effectiveness for one-year and three-year survival.

**Fig. 3:**
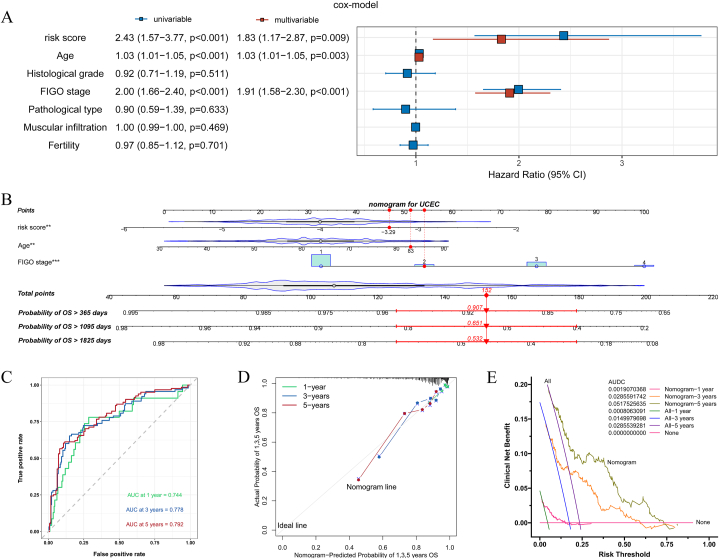
Construction and validation of the nomogram prognostic prediction model. (**A)** Univariate and multivariate Cox regression analysis of OS for the risk score and clinical factors; **(B)** Ordinary nomogram model. After the operation of UCEC patients, the “risk score” and “FIGO stage” were obtained through pathological analysis and genetic testing, combined with “Age,” and then these three predictive factors are applied to the nomogram model to obtain the exact probability of 1-year, 3-year, 5-year survival prognosis of UCEC patients; **(C)** ROC curves of 1-year, 3-year, and 5-year survival prognosis of UCEC patients based on the nomogram prognosis prediction model. The AUC value represents the degree of discrimination. AUC >0.7 indicates that the model has moderate predictive performance; **(D)** Calibration curves of 1-year, 3-year, and 5-year survival prognosis of UCEC patients based on the nomogram prognosis prediction model. The closeness of the model line (nomogram) to the ideal line (diagonal) represents the degree of calibration. The closer the model line is to the ideal line, the closer the probability predicted by the model is to the real probability, that is, the more ideal the model; **(E)** DCA curves of 1-year, 3-year, and 5-year survival prognosis of UCEC patients based on the nomogram prognosis prediction model. The AUDC value represents the net clinical benefit. The model has about twice the AUDC (net clinical benefit) compared to “All” at each time point.

It was evident that the LASSO Cox-based “risk score,” which encompassed four crucial prognostic PRGs, possessed superior predictive value compared to individual genes. However, its predictive capacity remains constrained. Consequently, we endeavored to enhance its predictive performance by developing a nomogram prognostic prediction model. The univariate and multivariate Cox regression analyses of clinical factors, in conjunction with the “risk score” depicted in Fig. 3A, yielded superior results compared to those presented in Fig. 1G. Consequently, the predictors for the nomogram were identified as “risk score,” “age,” and “FIGO stage.” The resulting prognostic prediction model, in the form of an ordinary nomogram, was illustrated in Fig. 3B. Following the surgical procedure on a patient with UCEC, the “risk score” and “FIGO stage” can be determined through pathological analysis and genetic testing. In Fig. 3B, the values of the three predictors were indicated in the appropriate locations on the nomogram, along with their corresponding “Age” values. Subsequently, a vertical dotted line was drawn to intersect with the “Points” line, resulting in the determination of 3 “Points.” These points were then summed to obtain the corresponding “Total points,” which were subsequently marked. Another vertical line was drawn to intersect with the “Probability of OS > 365, 1095, 1825 days” line below. Ultimately, this process yielded three precise probabilities for 1, 3, and 5-year survival. Consequently, informed clinical decisions could be made based on these outcomes. In this study, a patient diagnosed with UCEC with a “risk score” of −3.29, an age of 83 years, and a FIGO stage of level 2 was utilized as an illustrative case for the nomogram prognosis prediction model. The analysis revealed that the patient's total points on the nomogram were 152, indicating a corresponding survival probability of 0.907 over a period of 1 year (365 days), 0.651 over 3 years (1095 days), and 0.532 over 5 years (1825 days). The precise probability value proved to be highly advantageous in facilitating clinical decision-making and enhancing communication between healthcare professionals and patients. Moreover, it held significant implications for clinical transformation.

3. Validation and promotion of the nomogram prognostic prediction model

After the development of the nomogram prognosis prediction model, efforts were made to validate its predictive accuracy and scientific validity through a range of rigorous scientific validation methods. The ROC curve depicted in Fig. 3C demonstrates that the nomogram prognosis prediction model exhibits favorable medium predictive performance and discrimination in assessing the prognosis of UCEC patients at 1 year, 3 years, and 5 years post-operation (with AUC values of 0.744, 0.778, and 0.792, respectively). The calibration curve depicted in Fig. 3D demonstrates the favorable alignment between the nomogram-predicted probability and the actual probability for the prognostication of UCEC patients at 1 year, 3 years, and 5 years post-surgery. The model line closely adheres to the ideal diagonal line, signifying an optimal calibration and indicating the efficacy of the nomogram prognosis prediction model. The DCA curves depicted in Fig. 3E indicate that the “nomogram” (with AUDC values of 0.0019 at 1 year, 0.0286 at 3 years, and 0.0518 at 5 years) exhibited approximately twice the AUDC compared to the “All” group (with AUDC values of 0.0008 at 1 year, 0.0150 at 3 years, and 0.0286 at 5 years) across a significant range of decision thresholds for predicting the prognosis of UCEC patients at 1 year, 3 years, and 5 years post-surgery. This finding suggests that the nomogram prognosis prediction model offers superior clinical net benefit.

**Fig. 4:**
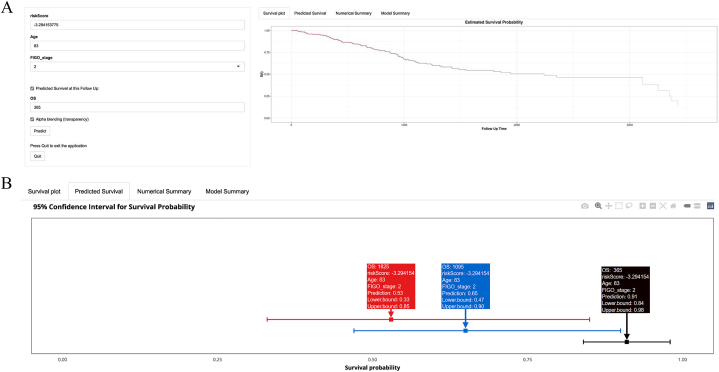
The web-based dynamic nomogram. **(A)** The nomogram could be accessed through a network device at any location. The specific link is: (https://shibaolu.shinyapps.io/DynamicNomogramForUCEC/). On the left was the parameter input box, which could load the postoperative information (“risk score”, “age,” and “FIGO stage”) of UCEC patients into the dynamic nomogram, and could also set a specific target survival time for prediction. On the right was the “Survival plot,” which could show the predicted specific survival curve of this patient; **(B)** The “Predicted Survival” box, which could display the individualized survival probability of the specific patient for the target survival time set in the “parameter box.” The same example as in Fig. 3B was also used here as a demonstration.

Following the successful validation of the conventional nomogram prognosis prediction model, an endeavor was made to develop a web-based dynamic nomogram and investigate its potential and significance for dissemination. The web-based dynamic nomogram, accessible to any country, region, or medical institution, can be utilized via the Internet by visiting the specific link (https://shibaolu.shinyapps.io/DynamicNomogramForUCEC/). In Fig. 4A, similar to the conventional nomogram depicted in Fig. 3B, relevant data such as the “risk score,” “age,” and “FIGO stage” of patients following UCEC can be entered into the parameter box located on the left-side. Subsequently, by clicking the “Predict” button, the distinct survival curve of the patient can be generated within the designated “Survival plot” box on the right-side. Moreover, the ability to display the survival probability for a patient at a specific time can be achieved by establishing distinct target survival times within the parameter box, as indicated by the “Predicted Survival at this Follow Up” button. This information is then presented in the “Predicted Survival” box on the right side of Fig. 4B, where the predicted outcomes for durations of 365 days, 1095 days, and 1825 days are showcased. Similar to Fig. 3B, the example provided continues to focus on a patient diagnosed with UCEC, possessing a “risk score” of −3.29, an age of 83 years, and a “FIGO stage” of level 2.

Hence, the nomogram prognosis prediction model exhibits favorable discrimination, calibration, and clinical net benefit in forecasting the prognosis of UCEC patients. Additionally, the web-based dynamic nomogram demonstrates high intuitiveness, accuracy, interactivity, personalization, and ease of implementation, thereby facilitating effective doctor-patient communication, clinical decision-making, and widespread adoption.

4. Experimental verification of the key PRG (GPX4) of the nomogram prognosis prediction model

After developing a conventional and online dynamic nomogram prognosis prediction model and assessing its scientific and promotional significance, we endeavored to substantiate the significance of key PRGs in UCEC through a sequence of well-established and rigorous experiments.

**Fig. 5:**
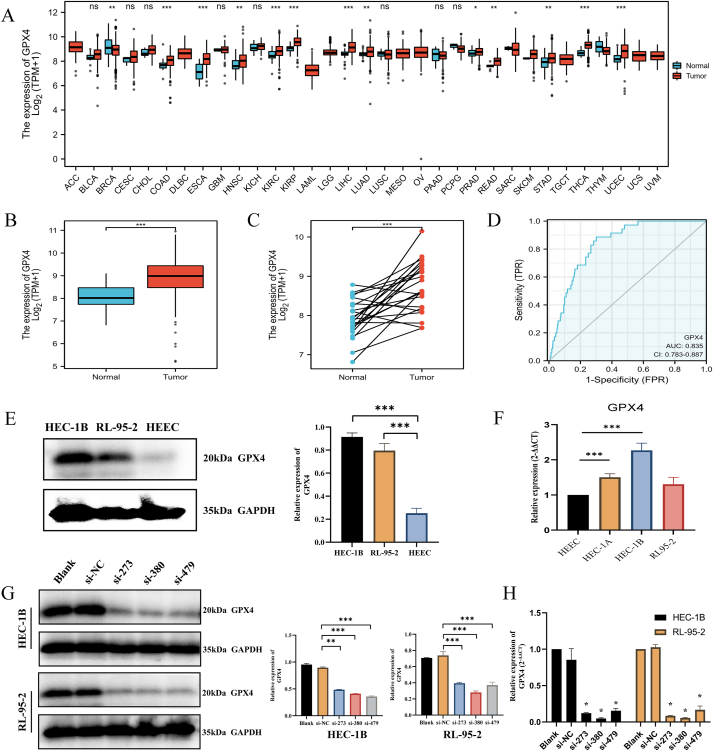
Expression levels and knockdown of the key gene GPX4. **(A)** Expression level of GPX4 in pan-cancer from TCGA database; **(B)** Differential expression of GPX4 in endometrial cancer and normal endometrium. **(C)** Differential expression of GPX4 in endometrial cancer and paired normal endometrium. **(D)** The ROC curve and AUC of GPX4 in the diagnosis of endometrial cancer. **(E)** Western blot of GPX4 protein expression in normal endometrial cell lines (HEEC) and endometrial cancer cell lines (HEC-1B, RL95-2). **(F)** RT-qPCR of GPX4 nucleic acid expression in normal endometrial cell lines (HEEC) and endometrial cancer cell lines (HEC-1A, HEC-1B, RL95-2). **(G)** Western blot of GPX4 knockdown validation in HEC-1B and RL95-2 cell lines after cell transfection. **(H)** Knockdown validation RT-qPCR of GPX4 in HEC-1B and RL95-2 cell lines after cell transfection. Among them, the original images of Western Blot in Fig. 5E and G were recorded in and could be referred to Supplementary Material 3.

Initially, Fig. 5A–C demonstrated that, utilizing TCGA data, GPX4 exhibited elevated expression levels in diverse tumor tissues compared to normal tissues, particularly in the case of UCEC. Furthermore, Fig. 5D indicated that the expression level of GPX4 possessed a moderate predictive capacity for diagnosing UCEC. Moreover, the analysis presented in Fig. S3A demonstrated that, according to the Human Protein Atlas (HPA), the protein expression of GPX4 was significantly elevated in tumor tissues compared to normal tissues.

Subsequently, the Western blot and RT-qPCR findings depicted in Fig. 5E and. F, and Fig. S3B revealed that the expression of GPX4 in UCEC cell lines was notably higher than that in normal control cells at both the nucleic acid and protein levels. These results further substantiated the viability of investigating the GPX4 gene as a potential research direction. Consequently, subsequent efforts were undertaken to silence GPX4 and assess its effects through a series of in vitro and in vivo experiments. Subsequently, the Western blot and RT-qPCR findings depicted in Fig. 5G and H demonstrated the effective knockdown of GPX4 by all three siRNAs. Among them, the original images of Western Blot in Fig. 5E and G were recorded in and could be referred to Supplementary Material 3.

**Fig. 6:**
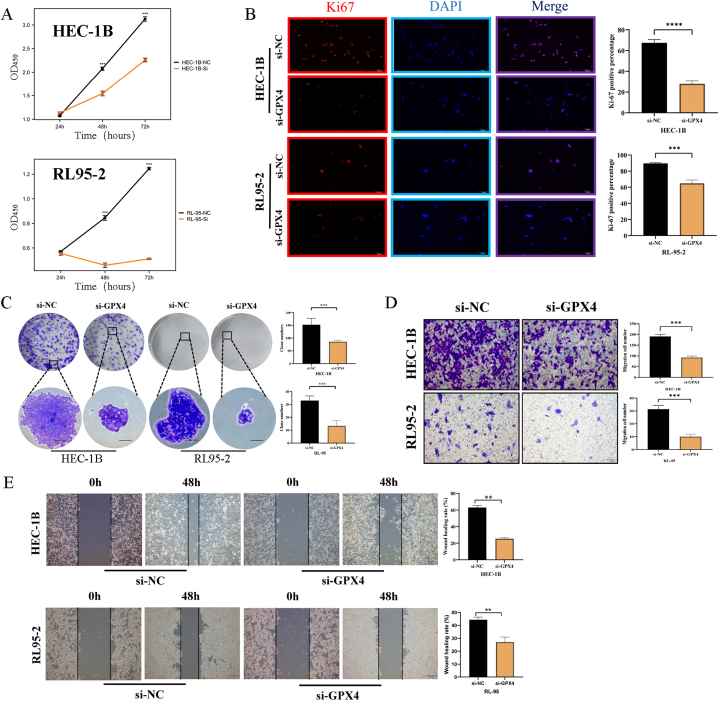
Effect of GPX4 knockdown on malignant biological behavior of UCEC cells (HEC-1B and RL95-2) after cell transfection. **(A)** CCK8 assay; **(B)** Ki-67 immunofluorescence; **(C)** Clone formation assay; **(A**–**C)** Results represent cell proliferation; **(D)** Transwell assay; **(E)** Wound healing assay; **(D**–**E)** Results represent cell invasion and migration.

**Fig. 7:**
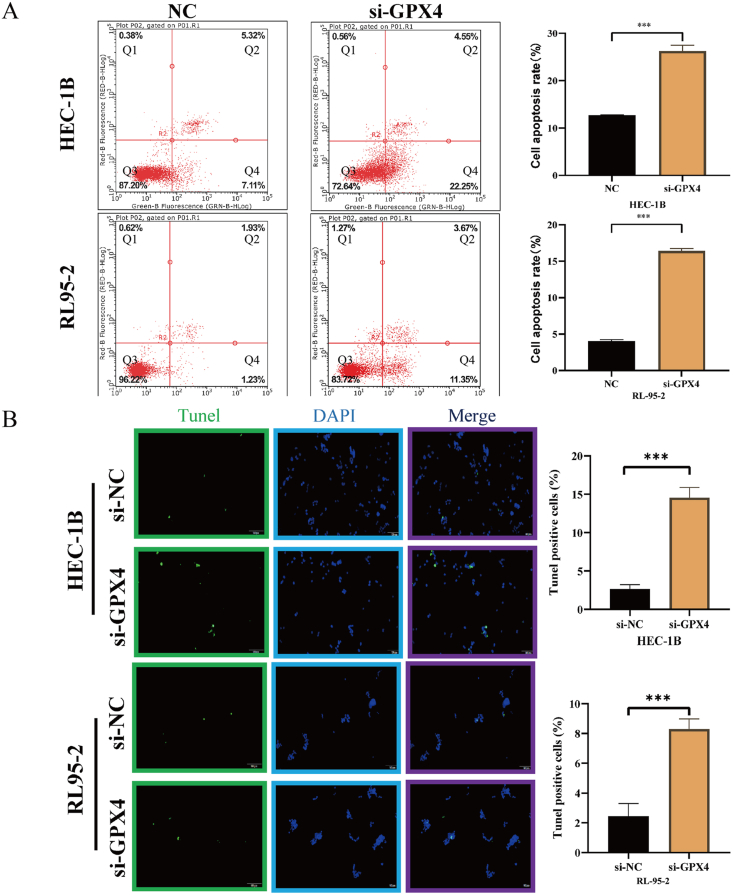
GPX4 knockdown induced apoptosis in UCEC cells (HEC-1B and RL95-2). (**A)** Characteristic fluorescence dot plots showing fluorescence-activated cell sorting (FACS) analysis as determined by annexin V staining and PI after knockdown of GPX4. The Q2 and Q4 regions represented apoptotic cells. The Q1 region represented dead cells. The Q3 region represented surviving cells; **(B)** TUNEL immunofluorescence staining of apoptotic cells after knockdown of GPX4. Green was TUNEL staining, blue was DAPI nuclear staining, and Merge was an overlay of the images. The graph showed the percentage of TUNEL-positive cells, representing cells in the apoptotic state. (For interpretation of the references to color in this figure legend, the reader is referred to the Web version of this article.)

Initially, the CCK8 assay revealed that the optical density of the si-NC group surpassed that of the si-GPX4 group at 24 h, 48 h, and 72 h in both HEC-1B and RL95-2 cell lines (Fig. 6A). Subsequently, the outcomes of the Ki-67 immunofluorescence assay exhibited a higher proportion of Ki-67 fluorescence-positive cells in UCEC cells within the si-NC group compared to the si-GPX4 group (Fig. 6B). The cloning assay revealed that the si-NC group exhibited a higher number of clones compared to the si-GPX4 group, as depicted in Fig. 6C. Consequently, these findings suggest that the proliferation of UCEC cells was hindered following GPX4 knockdown. Furthermore, the Transwell assay demonstrated that the si-NC group displayed a greater number of migrating cells in comparison to the si-GPX4 group, as shown in Fig. 6D. Additionally, the wound healing assay results indicated that, within UCEC cells, the wound healing rate of cells in the si-NC group was significantly higher than that of the si-GPX4 group after 48 h, as depicted in Fig. 6E. The aforementioned findings additionally substantiated the inhibition of UCEC cell migration following GPX4 knockdown. Flow cytometry experiments (Fig. 7A) revealed a lower percentage of apoptotic cells in the si-NC group compared to the si-GPX4 group, while the TUNEL immunofluorescence assay (Fig. 7B) further corroborated this outcome. Consequently, it can be inferred that the suppression of GPX4 enhanced the rate of apoptosis in UCEC cells.

Overall, in the context of the nomogram prognosis prediction model, GPX4 emerges as a significant oncogene in UCEC. Suppression of GPX4 impedes various biological activities of UCEC cell lines, including proliferation and migration, while concurrently inducing cell apoptosis.

5. Signaling pathway prediction based on the “risk score” of PRGs

Following the validation of GPX4 and “risk score” involvement in the nomogram prognosis prediction model in UCEC, we endeavored to anticipate and investigate its potential signaling pathways and mechanisms.

The findings from the GO, KEGG, and GSEA analyses presented in Fig. S4 indicate that the gene expression patterns observed in the “high-risk group” are predominantly associated with endometrial cancer, pathway in cancer, JAK STAT, natural killer cell mediated cytotoxicity, T cell receptor, B cell receptor, and other signaling pathways. These results not only validate the overall study to some extent, particularly in relation to signaling pathways such as endometrial cancer and pathway in cancer, but also suggest that investigating the regulation of immune cells and the immune microenvironment may be a promising avenue for future research. Subsequently, the findings depicted in Fig. S5, utilizing the EPIC, QUANTISED, MCPCOUNTER, and TIMER methodologies, indicated a significant association between the expression of the “high-risk group” and the extent of infiltration by diverse immune cells (including NK cells, macrophages, CD8^+^ T cells, and CD4^+^ T cells), as well as the expression of immune checkpoint markers (including LAG3, SIGLEC15, CTLA4, PDCD1LG2, CD274, and PDCD1).

Consequently, these four pivotal genes within the “risk score,” which hold substantial significance in the nomogram prognosis prediction model, may exhibit a close correlation with the regulation of the immune microenvironment. This aspect also represents a prospective avenue for future research. It is conceivable that a potential advancement can be achieved in this domain by employing the nomogram model to initially forecast the prognosis, subsequently enabling informed clinical decisions and facilitating the administration of personalized and rational immunotherapy for UCEC patients at high risk.

## 15 The knockdown of GPX4 impeded the growth of endometrial cancer graft tumors in nude mice

**Fig. 8:**
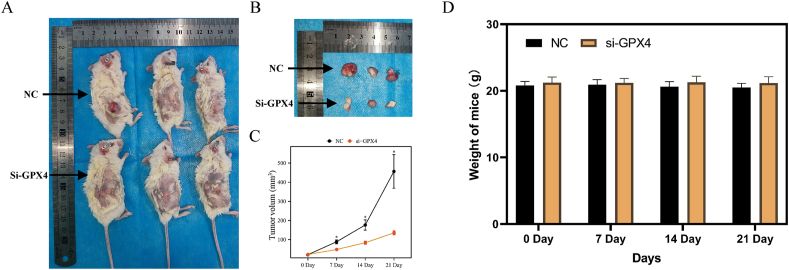
In vivo experiments in a murine xenograft model. (A) HEC-1B endometrial cancer cells, transfected with either negative control (NC) or si-GPX4, were intradermally injected into the ventral dorsum of C-NKG mice. **(B)** The tumors were excised on day 21, and the length diameter (L mm) and width diameter (W mm) of the tumors were assessed. **(C)** The growth curve of the transplanted tumors in C-NKG rats was plotted, with statistical significance denoted as *p < 0.05. **(D)** A histogram illustrating the changes in body weight of C-NKG rats before and after tumor seeding.

Fig. 8A presents a macrophotograph of a tumor-bearing mouse, while Fig. 8B showcases the excised tumors from the dorsal abdomen of mice. The Si-GPX4 group demonstrated smaller transplanted tumors in comparison to the NC group, as observed in the figure. Fig. 8C illustrates the peripheral volume growth curves of the tumors, indicating that the transplanted tumors in the Si-GPX4 group exhibited a slower rate of growth compared to those in the NC group. This disparity was determined to be statistically significant. Fig. 8D illustrates the lack of statistically significant changes in the body weight of mice. In general, the inhibition of GPX4 led to a noticeable suppression of transplanted tumor growth. In vivo experiments provided evidence that GPX4 played a role in promoting the proliferation of endometrial cancer transplanted tumors in mice.

## 16 Discussion

Accurate prognostication of UCEC is advantageous for subsequent clinical decision-making and enhancement of prognosis. The utilization of a nomogram clinical prediction model allows for an intuitive prediction of diagnosis or prognosis [27]. The dynamic nomogram based on web pages has stronger promotion value. Pyroptosis has demonstrated its pivotal involvement in tumorigenesis and prognosis across various human malignancies, such as lung cancer, liver cancer, colorectal cancer, cervical cancer, and leukemia [[28], [29], [30], [31]]. However, the perspective of individual genes has certain limitations. Therefore, the perspective of genetic risk models integrating multiple individual genes may lead to unique research discoveries. Consequently, this study aimed to develop a nomogram prognostic prediction model by identifying specific pyroptosis-related genes (PRGs) and relevant clinical features associated with prognosis. The predictive performance and utility of the model were subsequently validated, and the key gene (GPX4) was chosen for in vitro and in vivo basic experimental validation, ultimately providing a precise and widely applicable approach for prognostic evaluation of UCEC.

Initially, Fig. S6 and Fig. S7 served as comprehensive representations of the entire study, thereby facilitating researchers' comprehension of the research. The investigation was conducted utilizing the publicly accessible TCGA database, an expansive platform for global researchers. Numerous advancements in cancer research have been achieved by researchers leveraging the TCGA database. Nevertheless, it is imperative to acknowledge and value potential biases and limitations inherent in the research process. For instance, the TCGA database predominantly consists of Caucasian individuals, with a lesser representation of Asians or African Americans [32,33]. Then this study commenced by examining PRGs and identified four specific PRGs (GPX4, GSDME, NLRP2, and NOD2) that exhibited differential expression in UCEC and had prognostic implications (Fig. 1A–F). Additionally, the findings presented in Fig. S1 indicated a significant positive correlation between these key genes, particularly GPX4, MSI, and TMB. Besides, UCEC exhibited a high frequency of mutations that displayed a strong association with TMB and MSI [34]. Research findings have indicated that approximately 20 % of UCEC cases exhibit genetic alterations in MSI [35]. Furthermore, it has been observed that TMB is associated with both overall survival and the extent of immune infiltration in UCEC patients [36]. Additionally, in the context of these specific genes, GPX4 has been found to modulate the apoptosis of breast cancer cells by regulating mitochondria-mediated apoptosis, thereby influencing their resistance to drugs [37,38]. Moreover, the expression of the GSDME gene, which plays a crucial role in cellular demise, has been closely linked to the prognosis following chemotherapy [39]. The potential utility of the NLRP2 gene in prognosticating survival in individuals diagnosed with head and neck squamous cell carcinoma is evident [40]. Furthermore, NOD2 has been observed to enhance the susceptibility of hepatocellular carcinoma to chemotherapy through its targeting of the AMPK pathway and subsequent tumor inhibition [41]. These findings, along with other relevant investigations, suggest a potential close association between these four pivotal PRGs and the prognosis of UCEC.

However, in the second step of the study, the results obtained from the Cox analysis of PRGs in conjunction with clinical factors, as depicted in Fig. 1G, indicate that these four key PRGs cannot be employed directly and simplistically for the assessment of UCEC prognosis. This finding suggests an intricate correlation between the expression of these four key PRGs and the prognosis of UCEC, necessitating a more scientific and rational approach for analysis. LASSO Cox, known for its ability to analyze complex data and relationships, has demonstrated significant potential in research pertaining to the prediction of various diseases [42]. Subsequently, based on LASSO Cox, the four key PRGs were collectively fitted into the equation “risk score” = (−0.4323) * GPX4 + (0.2385) * GSDME + (0.0525) * NLRP2 + (−0.3299) * NOD2. The findings depicted in Fig. 2, Fig. S2, and Fig. 3A indicate that the “risk score” exhibits associations with several crucial clinical factors and has the potential to impact prognosis. Specifically, the comparison of the Cox of “risk score” with clinical factors in Fig. 3A to the Cox of PRGs with clinical factors in Fig. 1G highlights the superior prognostic value of the “risk score” over individual key PRGs. However, the ROC curve in Fig. 2F reveals that the predictive ability of the “risk score” for 1-year and 3-year prognosis remains insufficient (AUC <0.7).

In the third phase of the study, given the favorable performance of the nomogram in diagnosing and predicting the prognosis of various diseases [43], along with its user-friendly, precise, and easily disseminated nature, the decision was made to develop a dedicated nomogram clinical prediction model for assessing the prognosis of UCEC. This model would be based on PRGs, “risk score,” and clinical factors. Given the findings of multivariable Cox regression analysis, which demonstrated the significance of the “risk score,” “Age,” and “FIGO stage” as independent risk factors for UCEC, these three variables were employed as predictors in the development of a nomogram. The resulting nomogram, depicted in Fig. 3B, proved to be both straightforward and easily comprehensible. Most notably, it exhibited a high degree of accuracy in forecasting the survival probabilities of UCEC patients at 1 year, 3 years, and 5 years post-surgery. The provision of precise numerical data of this nature can alleviate the need for clinical practitioners to rely on imprecise and uncertain information during doctor-patient communication and clinical decision-making, thereby significantly contributing to the advancement of clinical transformation. Subsequently, the validation and construction of the nomogram prognostic prediction model hold equal significance [26]. The results depicted in Fig. 3C–E, namely the ROC, calibration curve, and DCA curve, demonstrated that the nomogram prognosis prediction model exhibited moderate diagnostic value, excellent calibration, and clinical net benefit in predicting the survival of UCEC patients at 1 year, 3 years, and 5 years post-surgery. The promotion and implementation of clinical prediction models are equally crucial as their development. Regardless of the accuracy of a constructed model, its impact will be limited to scientific literature unless it is widely promoted and adopted in clinical practice, thereby generating meaningful clinical value. Hence, we tried to develop a universally applicable network dynamic nomogram. Subsequently, the web-based dynamic nomogram (Fig. 4A and B) was made accessible to researchers worldwide via the internet (https://shibaolu.shinyapps.io/DynamicNomogramForUCEC/), facilitating widespread promotion. Moreover, this web-based dynamic nomogram not only enables the prediction and visualization of an individual patient's personalized survival curve, but also allows for the estimation of survival probabilities at any given time point, without being restricted to specific durations such as 1 year, 3 years, or 5 years. The clinical translation of clinical predictive models holds significant importance. In a practical clinical setting, following surgical intervention on a patient with UCEC, the pathologic tissue can undergo RNA sequencing. This sequencing process evaluates the expression levels of four crucial genes, enabling the calculation of a risk score. By inputting the key factors of risk score, age, and FIGO stage into the web page dynamic nomogram developed within this study, it becomes possible to obtain the patient's specific expected survival outcome. This outcome could include precise probabilities of survival at specific time intervals of 1, 3, and 5 years, thereby facilitating effective patient communication and aiding in clinical decision-making. Consequently, the nomogram clinical prediction model and web dynamic nomogram devised in this study have the potential to accurately forecast prognosis, facilitate doctor-patient communication and clinical decision-making, and ultimately accomplish clinical translation. Thus, the nomogram clinical prediction model, which incorporates PRGs, “risk score,” and clinical factors, exhibits favorable discrimination, calibration, predictive value, clinical net benefit, promotion value, and clinical transformation when predicting the prognosis of UCEC.

In the fourth step of research, it is recognized that scientific and rigorous basic experiments are crucial to ensure the reliability of the research. Despite the aggregation of the four key PRGs through the “risk score,” these four key PRGs still required thorough and comprehensive exploration on their own. Consequently, GPX4, which exhibited abnormal expression in various cancers and held significant weight in the “risk score,” was chosen as the starting point for further investigation. The findings from a series of in vitro experiments depicted in Figs. 5, Fig. 6, Fig. 7 indicate that GPX4 exhibits aberrantly high expression levels in UCEC cell lines. Moreover, the suppression of GPX4 has the potential to impede malignant biological activities, including the proliferation and migration of UCEC cells, while facilitating apoptosis. More importantly, the in vivo experimental results in Fig. 8 also suggested that knockdown of GPX4 could inhibit the growth of transplanted tumors. A prior investigation has demonstrated that the inhibition of GPX4 can effectively hinder the proliferation and colony formation of nasopharyngeal carcinoma cells [44]. Additionally, another study has revealed that the upregulation of GPX4 can facilitate prostate cancer metastasis through the inhibition of ferroptosis [45]. Furthermore, the results of GO, KEGG, GSEA, and immune comprehensive analysis depicted in Figs. S4 and S5 indicate a potential close association between the “risk score” derived from key PRGs and the immune microenvironment, including immune cell infiltration and immune checkpoints. It is widely acknowledged that the disruption of the tumor immune microenvironment significantly contributes to the initiation and progression of tumors [46]. Notably, the infiltration of immune cells, specifically CD8^+^ T cells and CD4^+^ T cells, may even serve as an independent protective factor for the prognosis of UCEC [47,48]. Furthermore, the efficacy of immune checkpoint inhibitor therapies has demonstrated promising outcomes [49]. These aforementioned findings and investigations not only validate the scientific rigor of this study, but also offer valuable insights and potential avenues for future research and advancements in immunotherapy.

Upon reflection of the comprehensive study, several noteworthy findings merit affirmation. The study adeptly devised, validated, and elucidated a prognostic prediction model based on PRGs for UCEC, employing a logical and scientific approach. Furthermore, a web-based dynamic nomogram was successfully constructed, showcasing its potential for widespread adoption. Of paramount significance, the study extensively investigated the role of the pivotal gene GPX4 in UCEC through meticulous in vitro and in vivo experiments. On the other hand, the present study employs the LASSO method to amalgamate the expression levels of four pivotal genes into a risk score, thereby obviating the reliance on a solitary gene's expression. This approach proves advantageous in prognostic assessment and offers potential insights for future investigations. Nevertheless, certain limitations persist in this study, such as the exclusive utilization of data from 542 UCEC patients sourced solely from the TCGA database. The inclusion of larger sample sizes from multiple centers would enhance the generalizability and value of the findings.

## 17 Conclusion

This study constructed and validated a prognostic prediction model, utilizing four key PRGs, for the personalized and intuitive prognosis assessment of UCEC patients. Additionally, a web-based dynamic nomogram was constructed, which holds significant promotional value. Notably, the key gene GPX4 exhibited potential oncogenic properties in UCEC, promoting malignant biological behavior and inhibiting apoptosis in its cells.

## Funding

This work is supported by PhD research startup foundation of the Third Affiliated Hospital of 10.13039/501100004605Zhengzhou University (BS20230104) and 10.13039/501100001809National Natural Science Foundation of China (No. 82372896 and No. 81872420).

## Ethics declarations

This study was reviewed and approved by [Animal Ethics Committee of Zhengzhou University], with the approval number: [No.20230318]. And the study has been carried out in accordance with either the U.K. Animals (Scientific Procedures) Act, 1986 and associated guidelines.

## Consent for publication

Not applicable.

## Data availability statement

The data can be obtained through the email under reasonable request: zmj230530@163.com.

## CRediT authorship contribution statement

**Lindong Zhang:** Formal analysis, Data curation, Conceptualization. **Jialin Wang:** Software, Formal analysis, Data curation. **Yan Guo:** Writing – review & editing, Writing – original draft, Funding acquisition. **Haodi Yue:** Validation, Supervision, Resources. **Mengjun Zhang:** Writing – original draft, Visualization, Validation, Supervision.

## Declaration of competing interest

The authors declare that they have no known competing financial interests or personal relationships that could have appeared to influence the work reported in this paper.

## List of abbreviations

Uterine Corpus Endometrial Carcinoma: (UCEC)
Pyroptosis-related genes: (PRGs)
Glutathione peroxidase 4: (GPX4)
Receiver Operating Characteristic curve: (ROC)
Area Under Roc Curve: (AUC)
Decision Curve Analysis: (DCA)
Area Under Decision Curve: (AUDC)
The Cancer Genome Atlas: (TCGA)
Tumor mutational load: (TMB)
Microsatellite instability: (MSI)
Gene Ontology: (GO)
Kyoto Encyclopedia of Genes and Genomes: (KEGG)
Gene Set Enrichment Analysis: (GSEA)
Tumor immune microenvironment: (TIME)
Tumor immune estimation resource: (TIMER)
