# Supplementary material for: The construction, validation and promotion of the nomogram prognosis prediction model of UCEC, and the experimental verification of the expression and knockdown of the key gene GPX4
Source: Heliyon. 2024 Jan 20;10(2):e24415. doi: 10.1016/j.heliyon.2024.e24415 (PMC10835249; doi:10.1016/j.heliyon.2024.e24415)
Supplement: Supplementary file 3 [file mmc3.pdf]

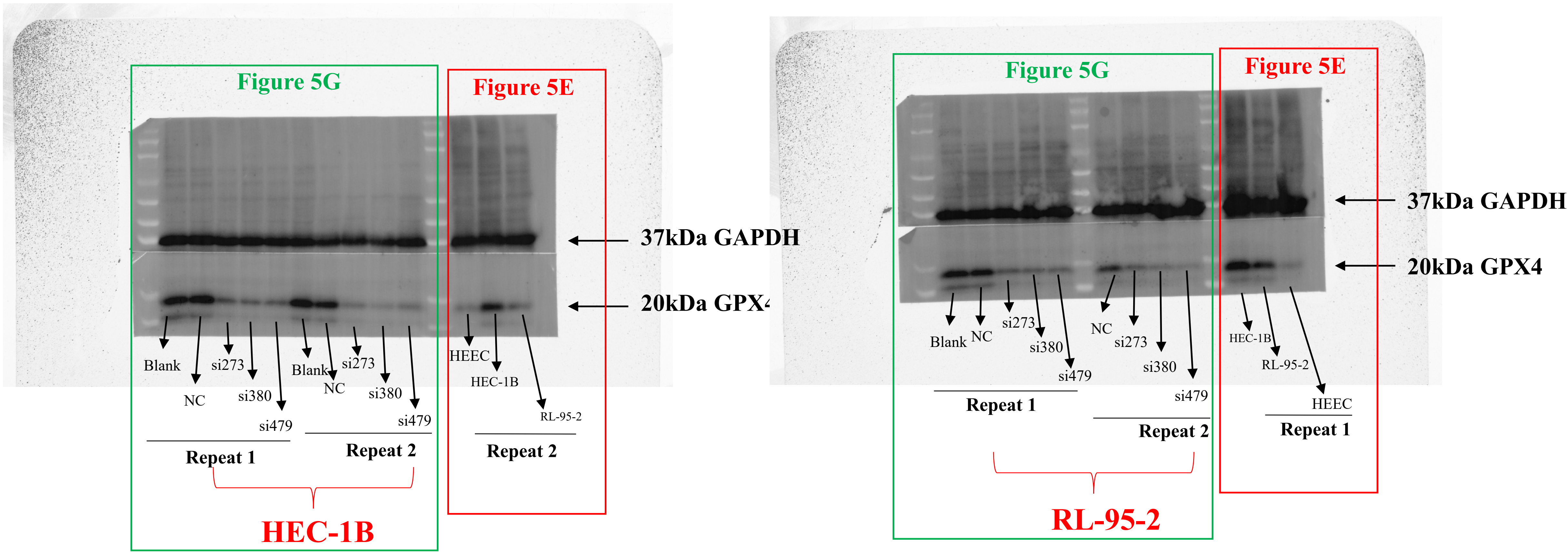

Figure 5G
Figure 5E
37kDa GAPDH
20kDa GPX4
Blank
NC
si273
si380
si479
Blank
NC
si273
si380
si479
HEEC
HEC-1B
RL-95-2
Repeat 1
Repeat 2
Repeat 2
HEC-1B
Figure 5G
Figure 5E
37kDa GAPDH
20kDa GPX4
Blank
NC
si273
si380
si479
NC
si273
si380
si479
HEC-1B
RL-95-2
HEEC
Repeat 1
Repeat 2
Repeat 1
RL-95-2

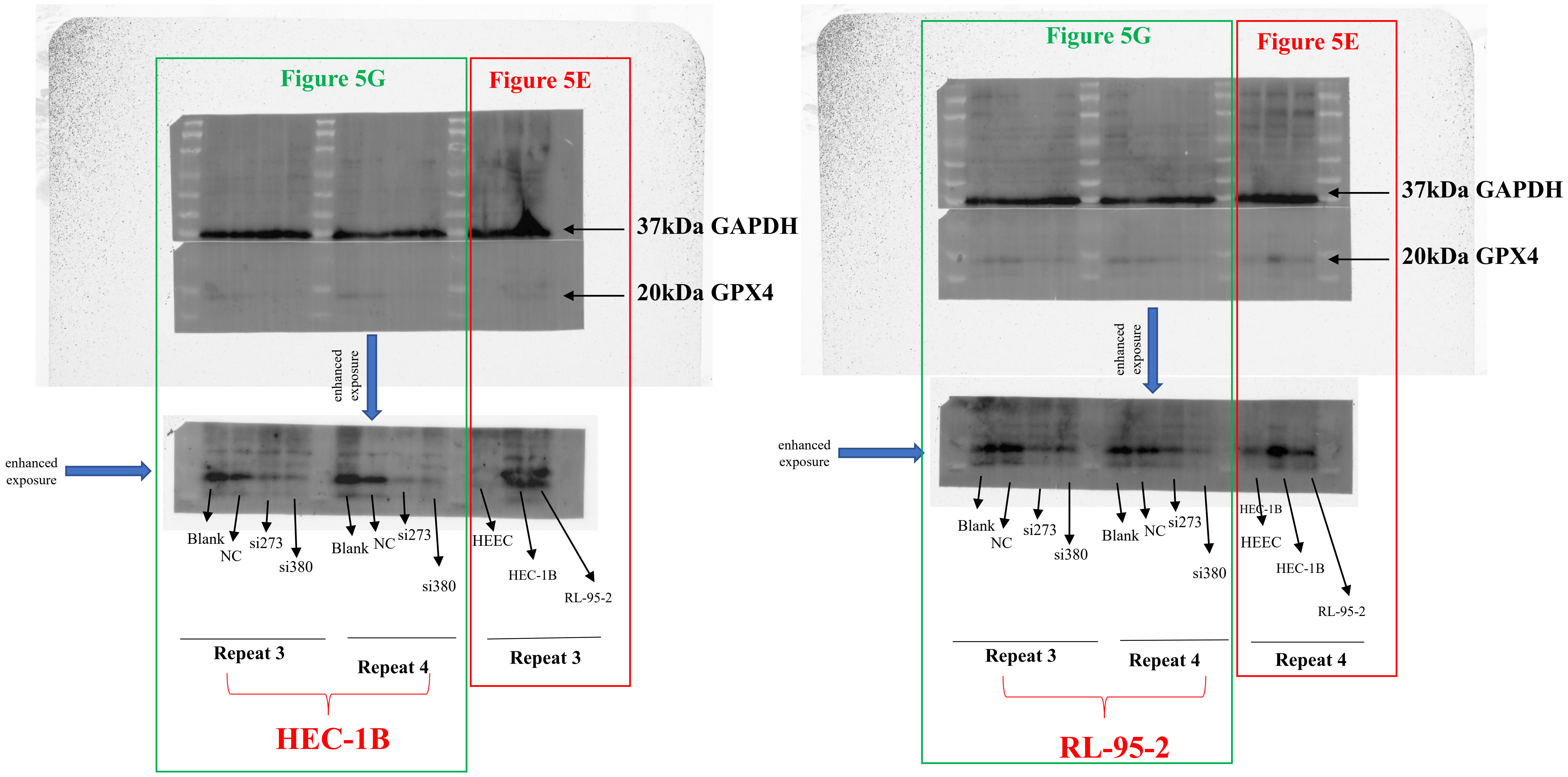

Figure 5G
Figure 5E
37kDa GAPDH
20kDa GPX4
enhanced exposure
enhanced exposure
Blank
NC
si273
si380
Blank
NC
si273
si380
HEEC
HEC-1B
RL-95-2
Repeat 3
Repeat 4
Repeat 3
HEC-1B
Figure 5G
Figure 5E
37kDa GAPDH
20kDa GPX4
enhanced exposure
enhanced exposure
Blank
NC
si273
si380
Blank
NC
si273
si380
HEC-1B
HEEC
HEC-1B
RL-95-2
Repeat 3
Repeat 4
Repeat 4
RL-95-2
