# Supplementary figures and images for: The construction, validation and promotion of the nomogram prognosis prediction model of UCEC, and the experimental verification of the expression and knockdown of the key gene GPX4
Source: Heliyon. 2024 Jan 20;10(2):e24415. doi: 10.1016/j.heliyon.2024.e24415 (PMC10835249; doi:10.1016/j.heliyon.2024.e24415)

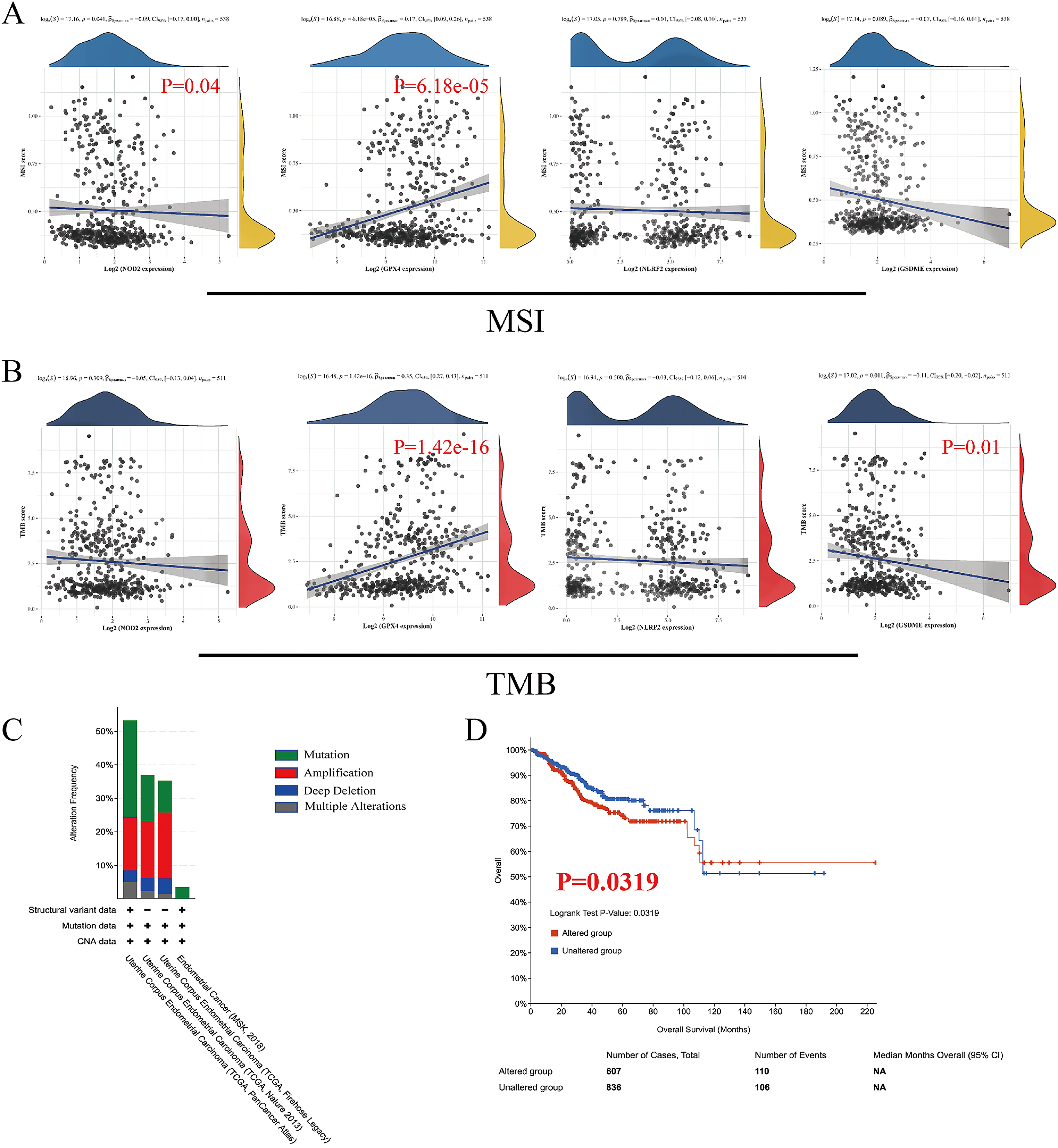

Supplement: Supplementary file 4 [file mmcfigs1.jpg]

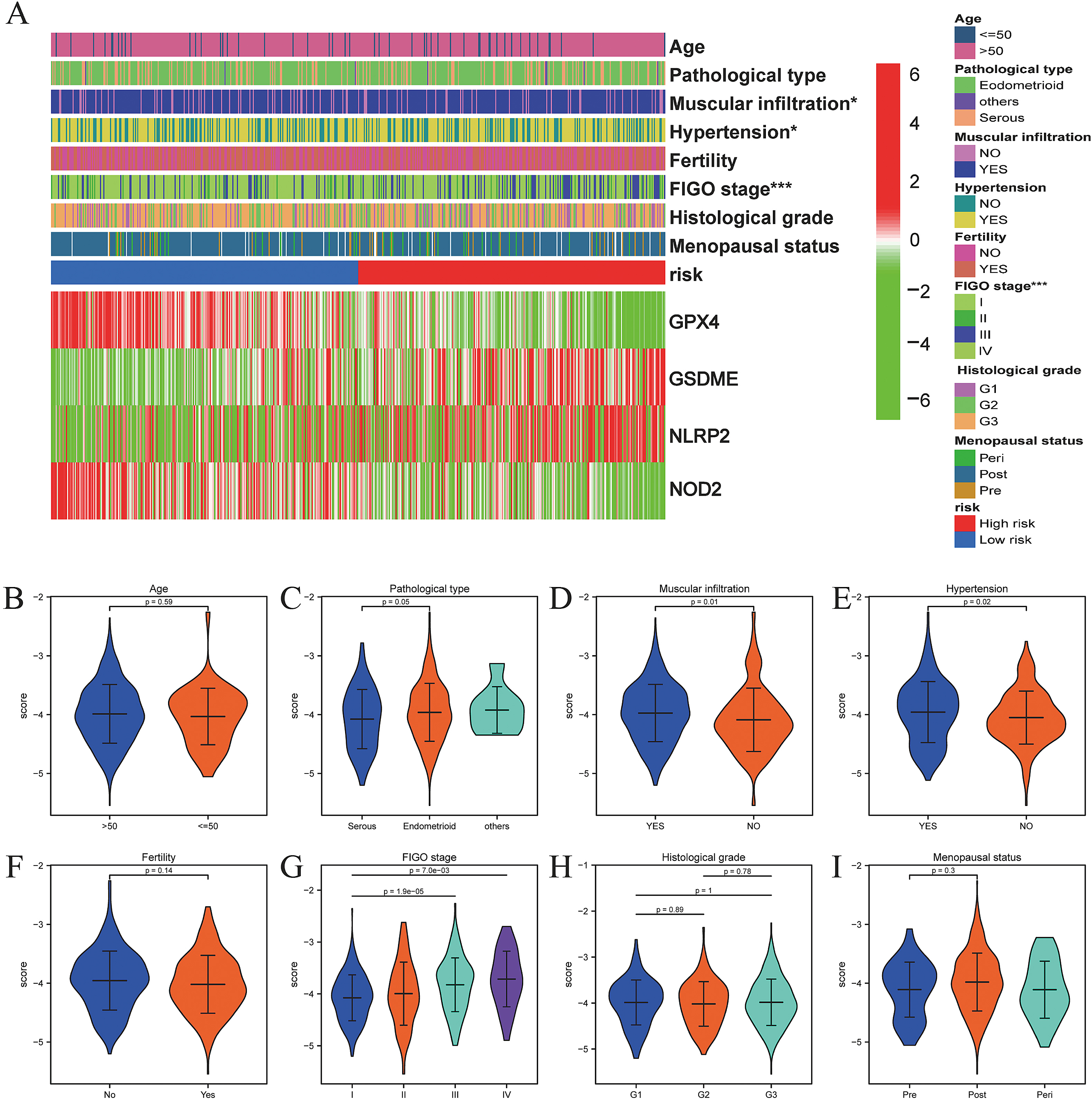

Supplement: Supplementary file 5 [file mmcfigs2.jpg]

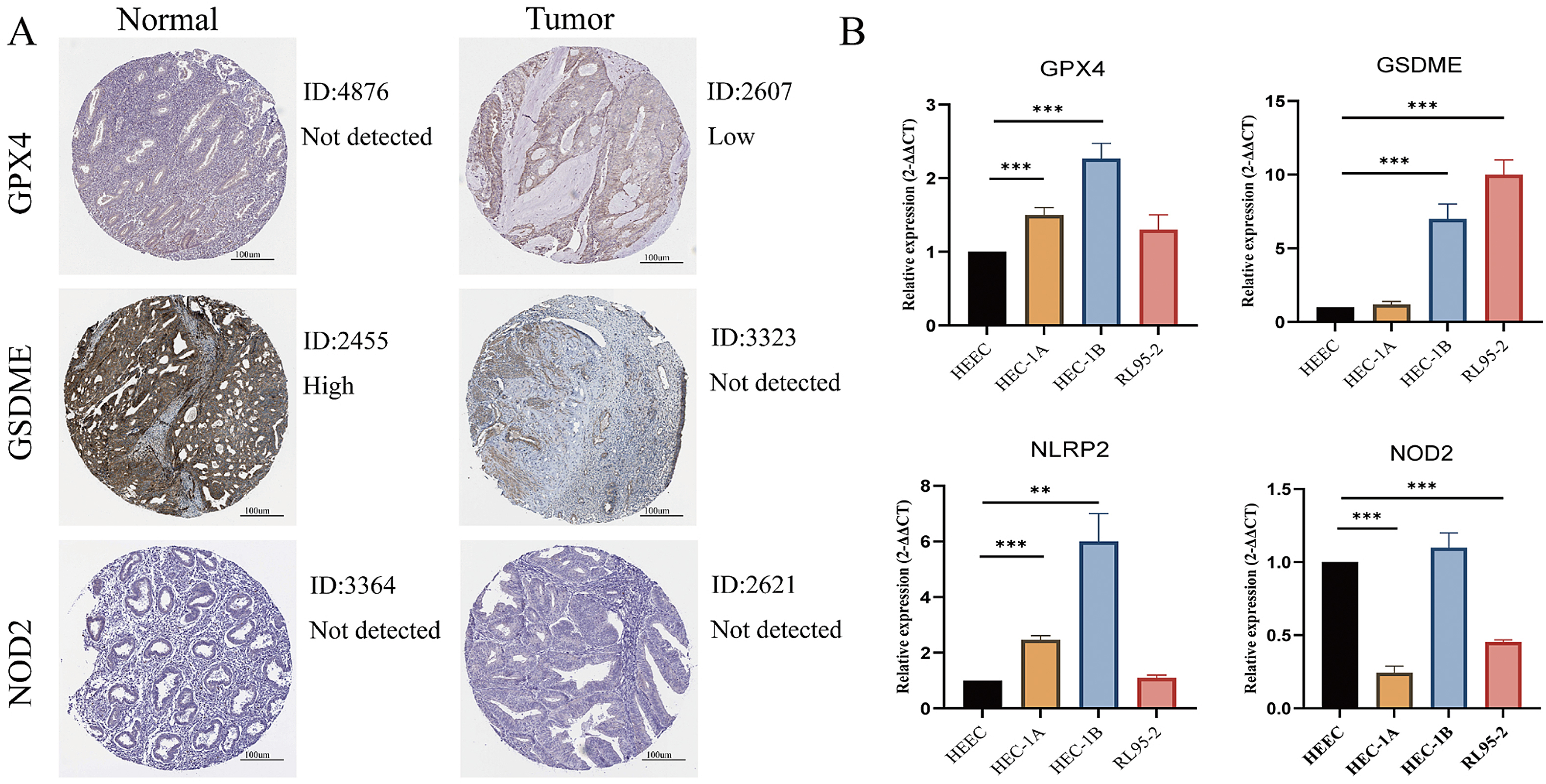

Supplement: Supplementary file 6 [file mmcfigs3.jpg]

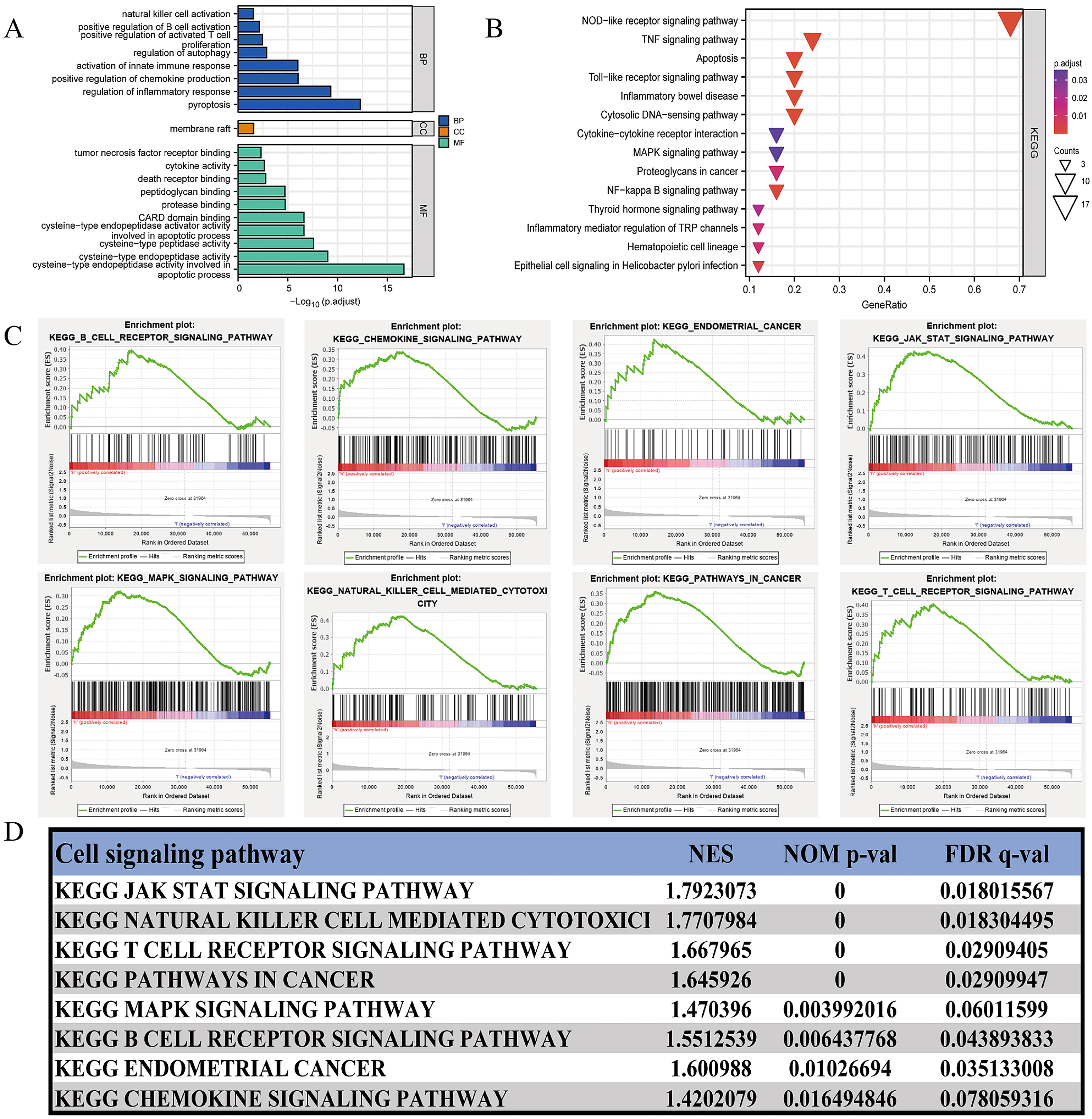

Supplement: Supplementary file 7 [file mmcfigs4.jpg]

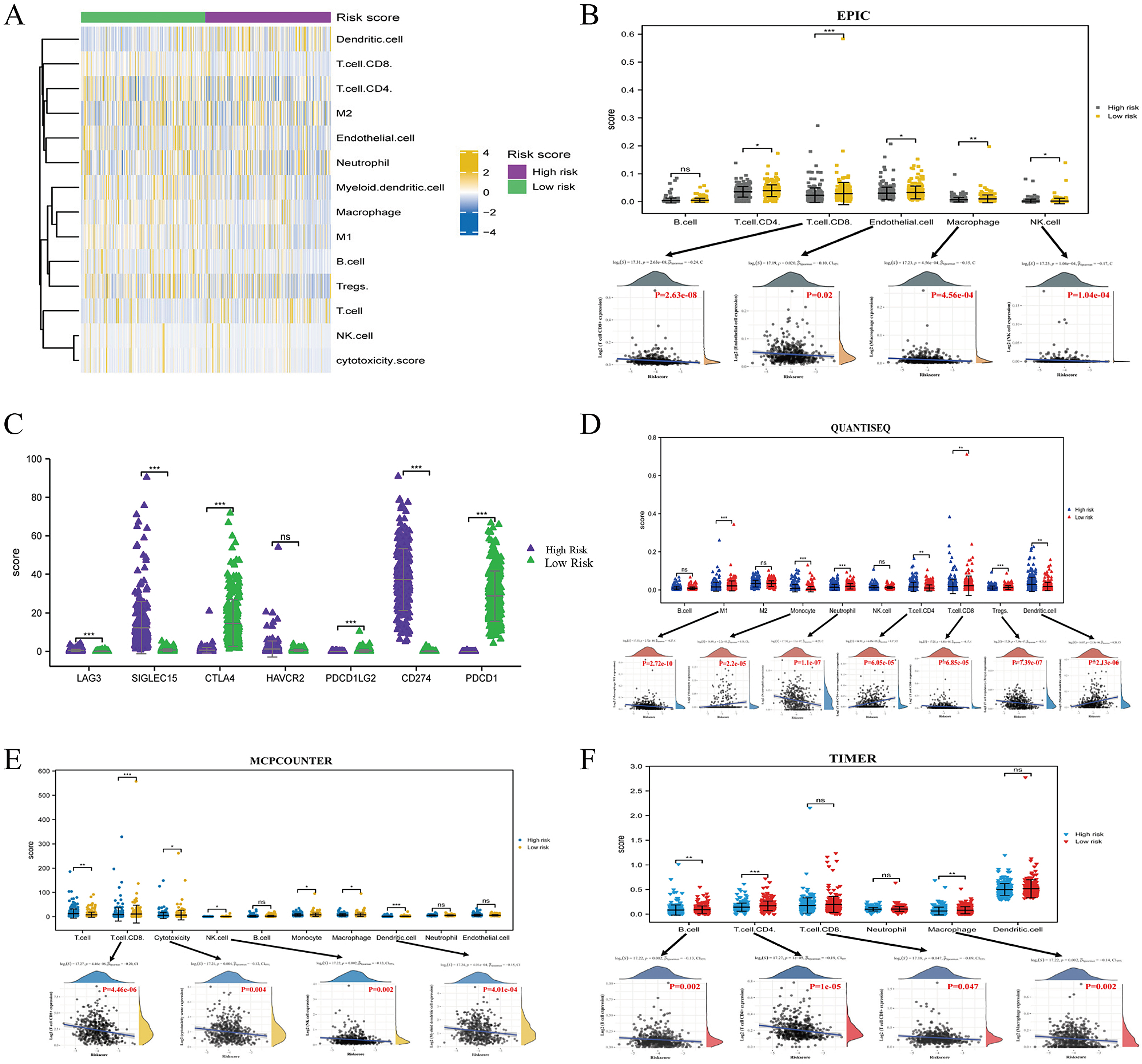

Supplement: Supplementary file 8 [file mmcfigs5.jpg]

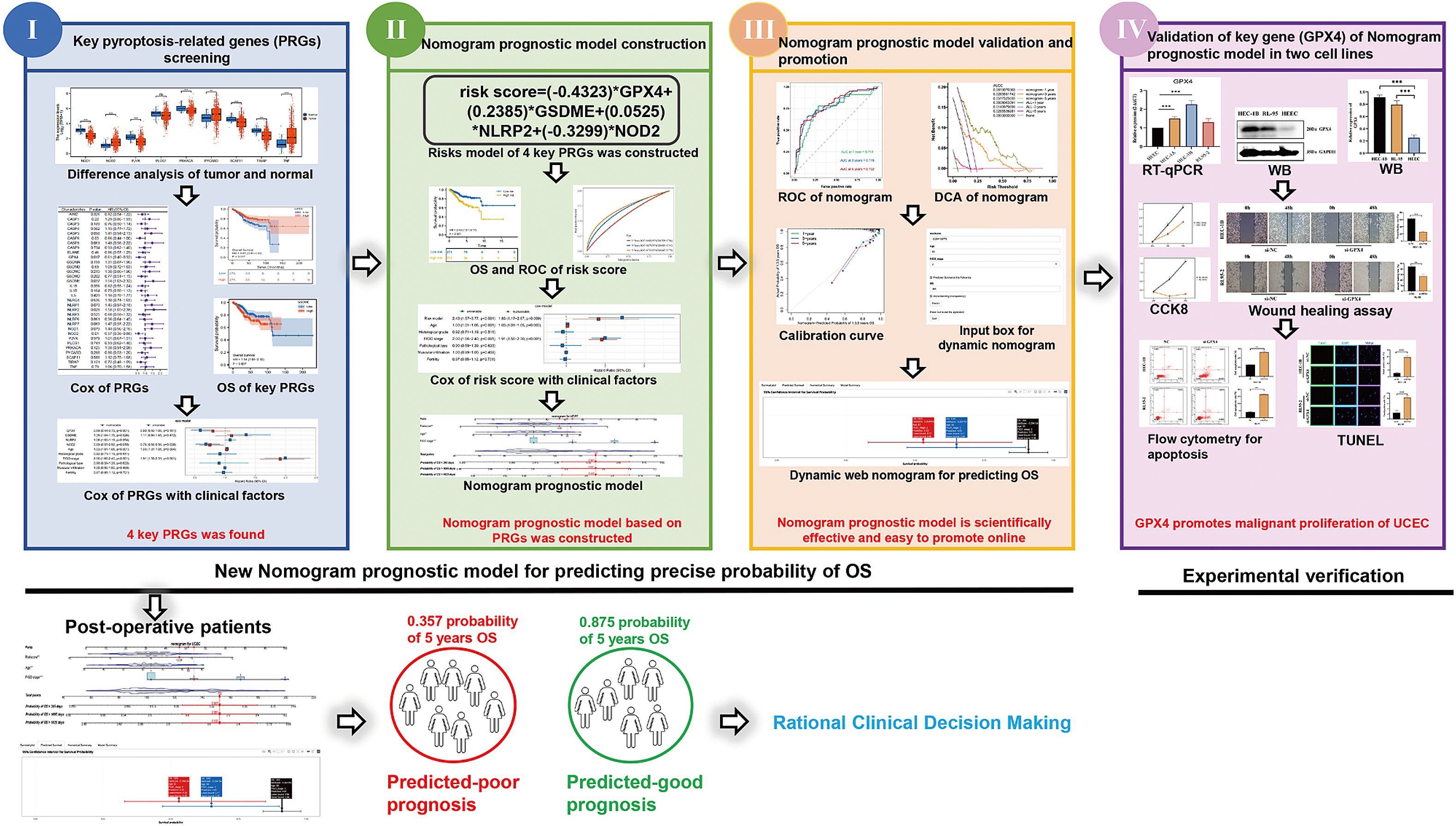

Supplement: Supplementary file 9 [file mmcfigs6.jpg]

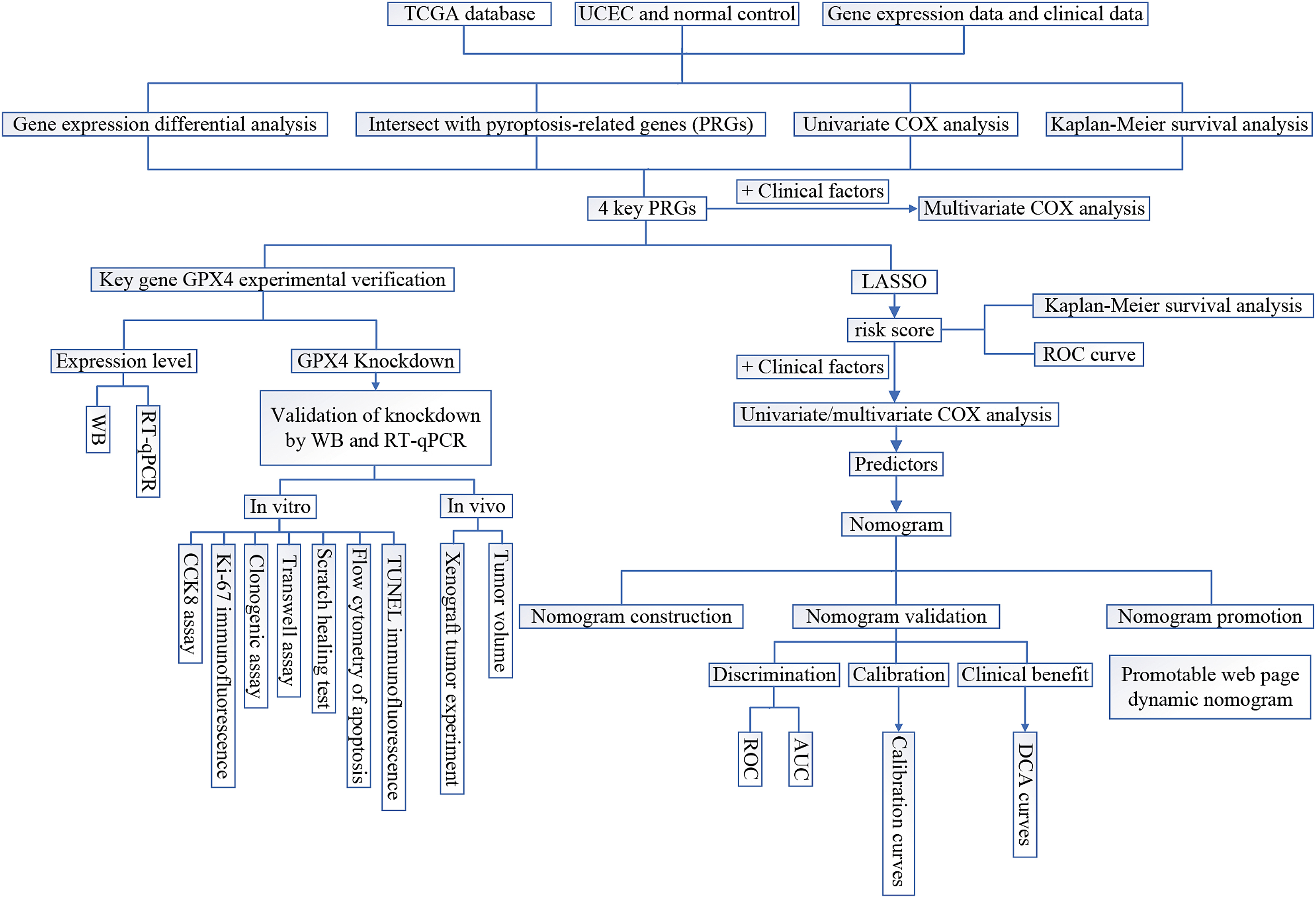

Supplement: Supplementary file 10 [file mmcfigs7.jpg]
